# A Flop Extraordinary

**Published:** 1896-06

**Authors:** 


A Flop Extraordinary. Is there a bug under the chip? The *Texas Medical News* (late *Texas Sanitarian*), J. W. McLaughlin and T. J. Bennett editors (McLaughlin, *loquitur*), says (page 315):

"* * * But aside from depriving the State Medical Association of naming those whom they desise to represent them on the board [not *unlikely* the getters-up and promoters of this mongrel bill. See?—Ed.] that the suggested changes [cutting off the homeopathic clause] would do, there are other good reasons, outside of the constitutional requirement, why the different schools of medicine [sic] are justly entitled [mark well] to an equitable representation on the Board of Medical Examiners. The bill requires that all physicians, regular, homeopathic, and eclectic, not already licensed, must successfully pass the examination of the board before they can practice medicine in this State; and as the three schools of medicine [mark well] hold quite different views of treatment [Well, I should say so. Here is a remark worthy of a great mind] the injustice of requiring the same examination of all applicants is apparent; it would not only be unjust, but it would create a flagrant monopoly that no member of our State Association would approve." [ ! ! ! ! ]

Just such a "monopoly" as the State Medical Association *for years* has endeavored to secure through committees on which Dr. McLaughlin has been, more than once, a member, and been defeated by these fellows every time, for no bill has ever before emanated from the State Association which provided for homeopathic representation; and yet, when the homeopaths and members of the legislature accused us of seeking a "monopoly," the charge has been spurned, and by none more vehemently than by the said McLaughlin.

Now, the *Texas Medical News* (late *Texas Sanitarian*), J. W. McLaughlin and T. J. Bennett editors, comes out unblushingly and champions the cause of homeopathy—advocates giving them this open recognition, and urges their "claims to consideration as factors in medical legislation."

What has caused such a complete change of heart, a flop most extraordinary? Let us see the record of these new converts to the homeopathic cause.

In March, 1891, pending the fight over the Association's bill before the legislature—a bill that asked for a straight board; when the homeopaths were crying, "they want a monopoly, give us equal representation," a certain pamphlet-address to the legislature, called "Homeopaths and Homeopathy Under the Calcium Light, their Claims to Consideration as Factors in Medical Legislation," was issued, signed *Wooten*, McLaughlin, Bennett and Daniel. From that pamphlet we extract a few sentences, as illustrating at once the "convenience" of the editor's "memory," and the magnitude of the somersault, or flop, just performed by the *Texas Medical News*. Remember, the following sentiments were subscribed to by Wooten, McLaughlin, Bennett and Daniel: * * *

"There are no sects in medicine; all the recent homeopathic outcry about there schools," and the "different systems of medicine" is the merest twaddle. Wooten, McLaughlin, Bennett and Daniel. * * *

"All 'sects' or 'schools' of medicine are mere 'pathies,' non-entities, and represent nothing,—not even a rational idea, and of all the ridiculous and absurd theories ever seriously proposed homeopathy is the most absurd and irrational." Wooten, *McLaughlin*, *Bennett* and Daniel. * * *

"This boastful, arrogant 'system' has not contributed one iota to the vast addition to scientific knowledge. It has done nothing but degrade the name of medicine, and bring reproach upon the profession." Wooten, *McLaughlin*, *Bennett* and Daniel. * * *

"Homeopaths want the people to believe that they practice only homeopathy, and insist that it is 'harmless,' and yet, on the sly, administer dangerous drugs in full doses; depart from the fundamental principle of homeopathy, and fly to the very opposite. Is this fair? is it rational? Is it honorable? When he does so, he necessarily abandons *in toto* the whole theory upon which the absurd practice of homeopathy is founded. What is he then? Surely not a homeopath; he is neither fish, flesh nor fowl; nor yet not even good red-herring. They would steal the livery of (the Hippocretean) heaven to serve the (Hahnemannian) devil in." Wooten, *McLaughlin*, *Bennett* and Daniel. * * * This looks, to a man up a tree, singularly like duplicity. Doing this thing, posing as homeopaths, and attempting to practice *medicine*, of which they are totally ignorant, in the great majority of cases, in the name of consistency and of common sense, upon what do they base their claim to be exempt from examination? [And *now*, to want a *medical* board to examine them, when they essay to practice *medicine*, would be "unjust" and a "flagrant monopoly," of which, etc., etc. Oh rare consistency; thou art indeed a jewel.] * * * If they essay the regular practice,—where does the homeopathy come in [and why shouldn't they be examined?] Wooten, *McLaughlin*, *Bennett* and Daniel. * * * "What, then, is the inevitable conclusion with reference to publicly declared homeopaths who openly confess that they do not confine themselves to homeopathy but practice all systems, varying their methods in accordance with the whims of their patrons? The only possible conclusion is that such men are imposters! There is no principle in their professional services, and they are physicians for revenue only. They are ready to resort to any trick or device likely to secure them victims. *Their sectarian title makes them quacks!*" Wooten, *McLaughlin*, *Bennett* and Daniel.

"Their sectarian title makes them quacks." Remember this a few minutes. On page 316, May No. *Texas Medical News*, Dr. McLaughlin says:

"The statement made that it is unethical for a regular to cooperate with a homeopath or eclectic on a board of medical examiners is, in my opinion, foolish. The duties of the board are purely executive, and we think it is no more unethical to serve with irregulars on a board of medical examiners than it is to serve with them on other executive boards,—school, church or municipal."

That is sheer sophistry. Dr. McLaughlin knows that association with irregulars on a medical board is professional, and in the other instances, social or political; and the above is the same as to say, "we think it is no more unethical to consult (professionally) with a homeopath than to confer with him socially or on business. The code makes the distinction in professional matters, but none in social, or business; and as the doctor has said of the homeopaths (*supra*), "their sectarian title makes them quacks," let us read it that way and see how it sounds;—"We think it no more unethical to consult professionally with a quack than we do to consult him on business or social matters."

The objection that to fraternize with a quack professionally on a medical board, "is unethical" may, in his opinion, be, as he says "foolish" [like a fool], and he may think the authors of and adherents to the code are—as he clearly intimates, fools; but we were not prepared to hear an ex-President of the Texas State Medical Association thus openly express his contempt for the code and its requirements.

It is alleged that expediency requires that Texas shall ask for a mixed board, because we have not heretofore been able to get an unmixed one; and that other States have done so. Two wrongs never made a right; and because Ohio and Louisiana have sacrificed honor and principle for expediency, it is no reason why Texas should do so. This *determination* to have a bill to regulate the practice at any cost, even of professional honor and dignity, must fix still more firmly in the minds of legislators the suspicion that a selfish motive must underlie the efforts. We have been accused of it, and we repeat, if we can not have legislation for the suppression of quackery without the assistance of *the principal element of quacks*—a law to shut off all *other* kind—why, let us cease all efforts in that direction. Such an alliance does look to a disinterested person as if the doctors are terribly distressed at the thought of the dear people suffering the dangers of—*all other kinds* of quacks except the homeopaths. Should we not endeavor to protect them from this fellow who poses as homeopath and attempts to practice medicine, of which he is ignorant?

It has been offered the homeopaths, if they will practice homeopathy, they will be exempt from examination, or else, let them have a board of their own. We can not see why they should not be examined by a medical board when they essay to practice *medicine*, any more than any one else, doing the same thing.

At the time the Association's bill to regulate the practice of medicine was disposed of, there was much confusion, and it was difficult to understand just what was done. The *Daily Gazette* (or *Mail*) stated that the bill was adopted with the hemeopathic clause stricken out. And as we took most of our notes from those of the reporters we so stated in our May issue. Dr. McLaughlin, in the *Medical News*, states that the bill was adopted without change in that respect. In this state of doubt and conflict we wrote the secretary for information, stating our recollection and the report of the *Gazette*. Just as we close, the following card was received which shows that neither of the two journals was exactly correct, but that the "Red Back" had been misinformed as to striking out the clause, and we make this statement in justice to the *News*. Dr. West says:

Galveston, May 31, 1896.

Dear Doctor:—The report of the Wilson Committee was adopted without material change or additions. What you refer to was a resolution, offered by Dr. Brittain, to the effect that homeopaths and eclectics were only recognized in the bill insofar as they are recognized by the constitution of the State. This resolution was explanatory and did not alter the bill in the slightest. Truly yours, H. A. W.

THAT RESOLUTION.—Since the above card was received the JOURNAL has been favored by Dr. Becton with a copy of that remarkable resolution; here it is, we forbear comment for the present:

"WHEREAS, The Texas State Medical Association adopted the report of the committee wherein it was recommended that the homeopaths and eclectics be recognized in the law, in order to get a constitutional law passed, governing the practice of medicine, and as some uninformed persons may construe this into an endorsement of the homeopaths and eclectics;

"Therefore, we positively declare that we do not recognize them only so far as they are recognized by statutes and constitution of the State of Texas to enable us to have a medical law passed in the State, but that we stand by the Code of the American Medical Association, and will expel any doctor of the State of the Texas Medical Association who will lower the dignity of regular medicine as to meet them in consultation."

